# Switching of Sox9 expression during musculoskeletal system development

**DOI:** 10.1038/s41598-020-65339-9

**Published:** 2020-05-21

**Authors:** Ryotaro Nagakura, Masahito Yamamoto, Juhee Jeong, Nobuyuki Hinata, Yukio Katori, Wei-Jen Chang, Shinichi Abe

**Affiliations:** 1 Department of Anatomy, Tokyo Dental College, 2-9-18 Misaki-cho, Chiyoda-ku, Tokyo 101-0061 Japan; 2 Department of Basic Science and Craniofacial Biology, New York University College of Dentistry, 345 E. 24th Street, New York, NY 10010 USA; 3 Department of Urology, Kobe University Graduate School of Medicine, 7-5-1, Kusunoki-cho, Kobe 650-0017 Japan; 4 Department of Otolaryngology-Head and Neck Surgery, Tohoku University Graduate School of Medicine, 1-1 Seiryo-machi, Aoba-ku, Sendai, Miyagi 980-8574 Japan; 5 School of Dentistry, College of Oral Medicine, Taipei Medical University, 250 Wu-Hsing Street, Taipei, 110 Taiwan

**Keywords:** Immunochemistry, Atomic force microscopy

## Abstract

The musculoskeletal system, which comprises muscles, tendons, and bones, is an efficient tissue complex that coordinates body movement and maintains structural stability. The process of its construction into a single functional and complex organization is unclear. SRY-box containing gene 9 (Sox9) is expressed initially in pluripotent cells and subsequently in ectodermal, endodermal, and mesodermal derivatives. This study investigated how Sox9 controls the development of each component of the musculoskeletal system. Sox9 was expressed in MTJ, tendon, and bone progenitor cells at E13 and in bone at E16. We detected Sox9 expression in muscle progenitor cells using double-transgenic mice and myoblastic cell lines. However, we found no Sox9 expression in developed muscle. A decrease in Sox9 expression in muscle-associated connective tissues, tendons, and bones led to hypoplasia of the cartilage and its attachment to tendons and muscle. These results showed that switching on Sox9 expression in each component (muscle, tendon, and bone) is essential for the development of the musculoskeletal system. Sox9 is expressed in not only tendon and bone progenitor cells but also muscle progenitor cells, and it controls musculoskeletal system development.

## Introduction

The musculoskeletal system comprises muscles, tendons, and bones. It is an efficient tissue complex that coordinates body movement and maintains structural stability^1^. This requires effective linkage of muscles to bones. Many studies have reported the development of each component of the musculoskeletal system, but the process of its construction into a single functional and complex organization is unclear.

Muscles, tendons, and bones differ in their developmental origins^2–8^, yet they interact during differentiation. Chen and Galloway^8^ reported that artificial rupture of the myotome impedes syndetome formation. In contrast, cranial tendon progenitor cells emerge without dependence on muscles, yet muscle presence is essential for their normal differentiation^8^. Huang *et al*^9^. analyzed tendon development in forearms and fingers and found that tendons can be divided into a distal module showing cartilage-dependent development and a proximal module showing muscle-dependent development. In addition, the mechanical stress of muscle contraction affects the morphology of cartilage and bone during development^10^. Previously, we found that mutual contact between muscles and the tendon-like structure immediately promotes the development of bones^11^ and that there is a morphological association between muscles and bones^12^. In summary, studies need to focus on the analysis of muscles, tendons, and bones as a single functional organ^13^ rather than focusing on each as an independent tissue component.

SRY-box containing gene 9 (Sox9) is a transcription factor essential for musculoskeletal system development. Sox9 is expressed in all cartilage progenitor cells and cartilage cells except hypertrophic chondrocytes^14,15^, and it plays a key role in a series of processes involved in endochondral ossification^16^. Tendons temporally express Sox9 during the early stage of development, but Sox9 is not expressed in developed tendon cells^17^. In a *Sox9*^*Cre*^ mouse cell lineage analysis, Sox9 was found to be expressed in a subset of tendon and cartilage progenitor cells^18,19^. Although a few studies reported high Sox9 expression in myoblastic cells *in vitro*^20–22^, it is unclear whether Sox9 is expressed in muscles *in vivo*.

In this work, we clearly identified that Sox9 is expressed in muscle progenitor cells *in vivo*. Since Sox9 is expressed in a subset of tendon and cartilage progenitor cells^17,18^, in this study, we investigated how Sox9 controls the development of each component of the musculoskeletal system.

## Results

### Connection between muscle progenitors and tendon-bone progenitors

**Figure 1:**
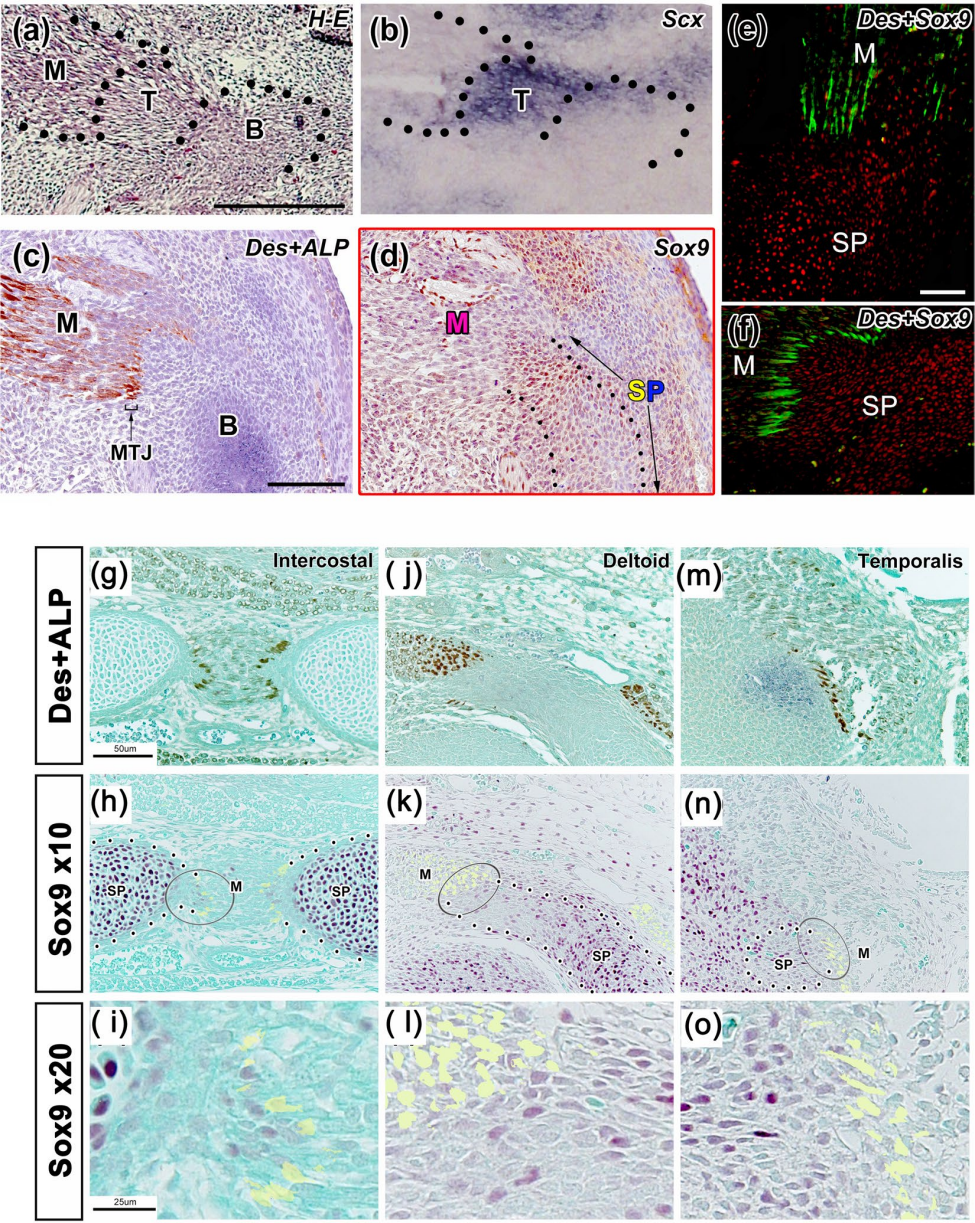
Sox9 expression in tendon and bone. (**a**–**d**,**f**) Sagittal plane images of the TMJ at E13.5 and (**e**) sagittal plane image of the triceps brachii muscle attachment to the ulna at E13.5. (**a**–**d**) Serial sections. (**a**) H&E staining; (**b**) in situ hybridization, Scx staining; (**c**) immunohistochemical staining of ALP and desmin; (**d**) immunohistochemical staining of Sox9; and (**e, f**) immunohistochemical staining of desmin and Sox9. (**g**–**o**) Sagittal plane images with immunohistochemical staining of (**g**, **j**, **m**) desmin and (**h**, **k, n**) Sox9. (**i, l, o**) Enlargements of (**h**, **k**, **n**), respectively. E13.5–E14.5 attachment regions of the (**g**–**i**) intercostal muscle to the ribs, (**j**–**l**) deltoid muscle to scapula, and (**m**–**o**) temporal muscle to coronoid process. The desmin-accumulating MTJ is in contact with Sox9^+^ progenitor cells. Scale bar = 50 μm (**a–f**, **g**, **h**, **j**, **k, m, n**) and 25 μm (**i**, **l**, **o**). M, muscle; T, tendon; B, condyle; SP, Sox9^+^ progenitor cells; Sox9, SRY-box containing gene 9; TMJ, temporomandibular joint; H&E, hematoxylin and eosin; ALP, alkaline phosphatase; MTJ, myotendinous junction.

Although Sox9 is observed throughout tendon and bone progenitors^17,18^, little is known about the connection between muscle progenitors and Sox9^+^ tendon-bone progenitors. To observe this connection, we performed immunofluorescence staining with antibodies against desmin, which is a marker of the myotendinous junction (MTJ)^11^, and Sox9, which is a marker of tendon and bone progenitor cells^17,18^. *In situ* hybridization of Scx^17,18^ and alkaline phosphatase staining^11^ allowed us to distinguish tendon progenitors from bone progenitors. We analyzed the connection at the presumed locations in five regions: the lateral pterygoid muscle attachment to the condyle of the mandible (Fig. 1a–d,f), the triceps brachii muscle attachment to the olecranon (Fig. 1e), the intercostal muscle attachment to the ribs (Fig. 1g–i), the deltoid muscle attachment to the scapula (Fig. 1j–l), and the temporal muscle attachment to the coronoid process of the mandible (Fig. 1m–o). The progenitor cells expressing Sox9 crossed from the tendon anlage to the bone anlage, and the most forward migrating cells made contact with the desmin-accumulating MTJ (Fig. 1).

**Figure 2:**
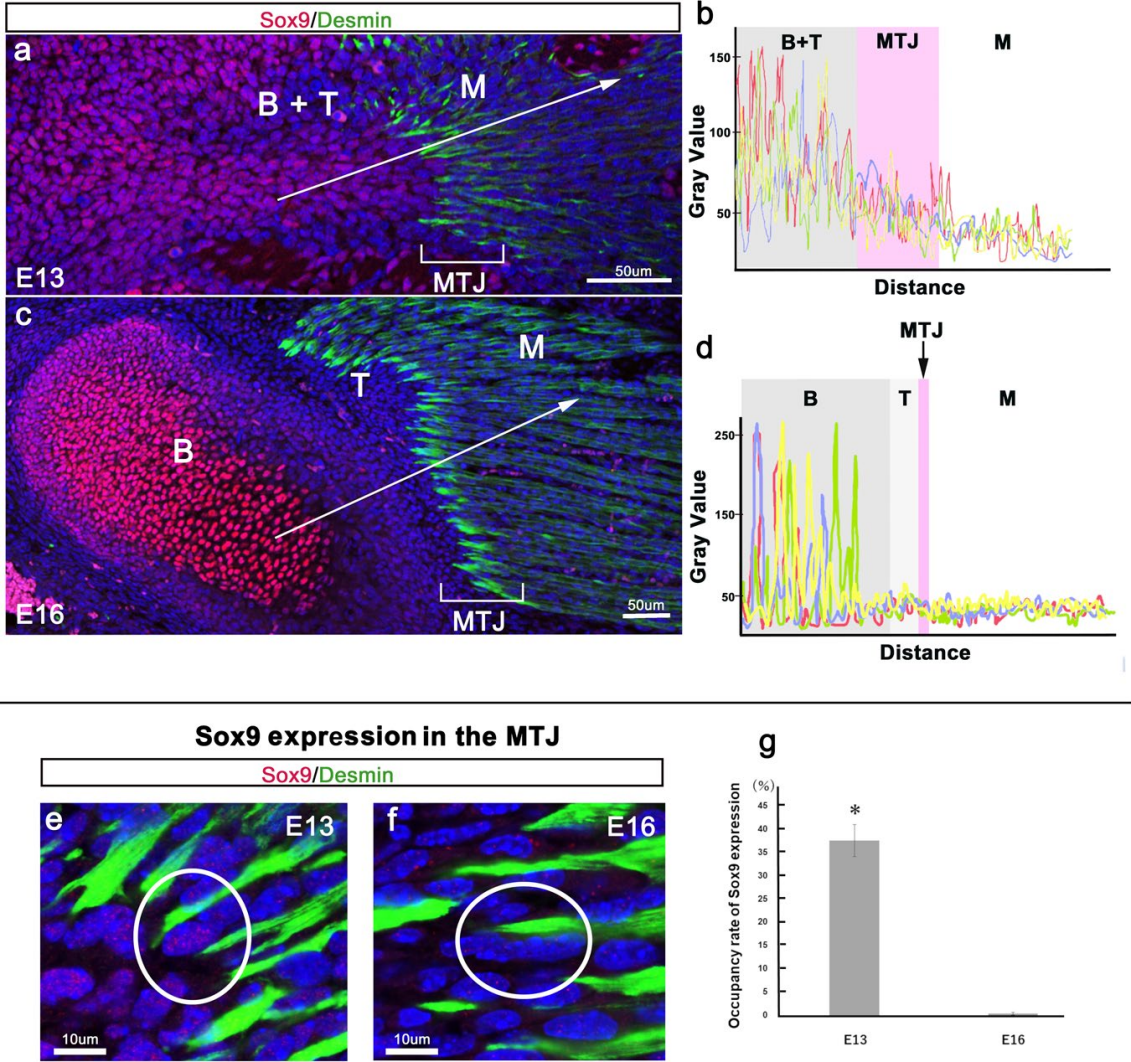
Switching of Sox9 expression during the formation of the musculoskeletal system. Sagittal plane images of the TMJ at (**a**) E13 and (**c**) E16. (**b**, **d**) Graph of the measured Sox9^+^ progenitor cell luminance. (**e**) High-magnification view of the MTJ in (**a**) and (**f**) high-magnification view of the MTJ in (**c**). (**g**) Occupancy rate of Sox9 expression. The fluorescence intensity vs. distance plot shows that Sox9 expression was identified in (**a**, **b**) future tendons, bone, and MTJ regions at E13.5 and in (**c**, **d**) cartilage cells at E16. The comparison of fluorescence intensity indicated that the intensity was>50 in the region of the MTJ at E13 but was <100 in the region of the MTJ at E16 (**b, d**). (**e–g**) The occupancy rate of Sox9 expression in the MTJ at E13 was high compared to that in the MTJ at E16 (*P < 0.05). Sox9, SRY-box containing gene 9; B, bone; M, muscle; T, tendon; MTJ, myotendinous junction.

Sox9 is essential for chondrocyte differentiation and cartilage formation^2^. It is temporally expressed in tendons during the early stage of development but not in developed tendon cells^17^. To clarify the role of Sox9 expression during tendon and bone development, we analyzed the fluorescence intensity of immunohistochemical staining. The fluorescence intensity versus distance plot showed switching of Sox9 expression. At E13, the fluorescence intensity was>100 in the tendon and bone regions (Fig. 2b). At E16, the fluorescence intensity was>100 in the bone region but <100 in the tendon (Fig. 2d). During detailed observation of the connection between muscle progenitors and tendon-bone progenitors, we noticed Sox9 expression in a part of the muscle. The fluorescence intensity of Sox9 expression was>50 in the MTJ region at E13 but <50 in the MTJ region at E16 (Fig. 2b,d). The occupancy rate of Sox9 expression in the MTJ at E13 was high compared to that in the MTJ at E16 (E13: 37.56 ± 6.02%, E16: 0.40 ± 0.45%, *P* < 0.05) (Fig. 2e–g).

These results demonstrated that Sox9 was expressed not only in the tendon and the bone but also in a part of the muscle: the MTJ (Fig. 2).

### Sox9 expression in muscle progenitor cells *in vivo*

**Figure 3:**
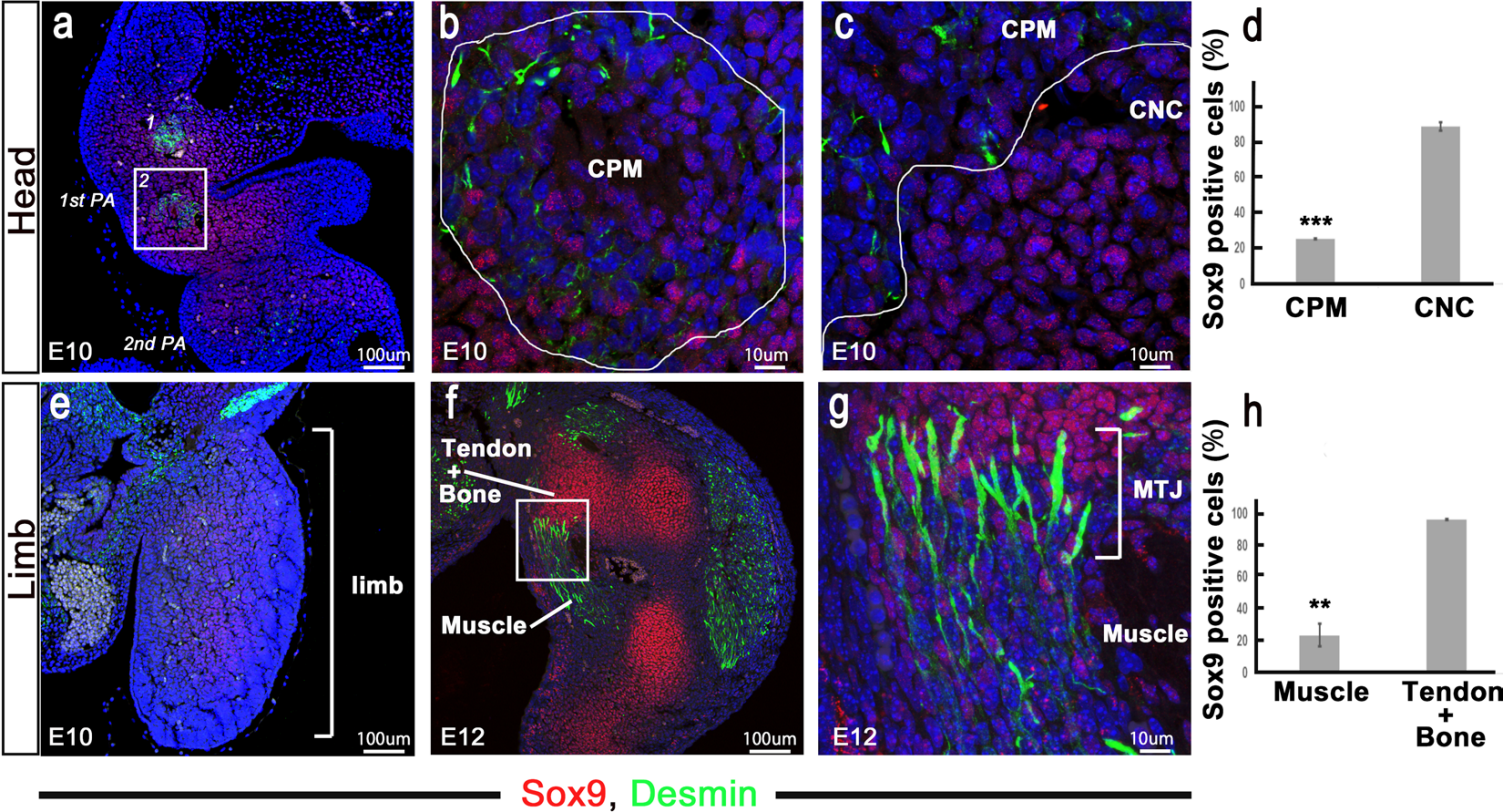
Sox9 expression in muscle. (**a–d**) Head at E10 and (**e-h**) limb at E10 and E12. All panels show immunohistochemical staining of desmin (green) and Sox9 (red). (**b, c**) High-magnification view of a square in (**a**) and (**g**) high-magnification view of a square in (**f**). (**d, h**) Comparison of Sox9^+^ progenitor of CNCs with those of the CPM. (**d**) The mass composed of muscle progenitor cells has few Sox9^+^ progenitor cells compared to CNCs (*P* < 0.001). (**h**) The limb muscle has few Sox9+ progenitor cells compared to the future tendon and bone (*P* < 0.01). Sox9, SRY-box containing gene 9; CNC, neural crest cell; CPM, cranial paraxial mesoderm; MTJ, myotendinous junction.

Some researchers have found Sox9 expression in myoblastic cells *in vitro*^20–22^. To determine whether Sox9 is expressed in muscle progenitor cells *in vivo*, we of double immunofluorescence staining with antibodies against desmin, which is a marker for muscle progenitors^11^, and Sox9^17,18^. In previous studies of head development^23,24^, we defined the cells in the desmin^+^ area as the cranial paraxial mesoderm (CPM), from which the head muscle originates (Fig. 3a–c). Cranial neural crest cells (CNCs), from which the head tendon and bone originate, were located in the superficial layer of each pharyngeal arch and were wrapped around muscle progenitor cells^23^ (Fig. 3a–c). At E10, we found two masses comprising desmin^+^ muscle progenitor cells in the first pharyngeal pouch (Fig. 3a). A mass of muscle progenitor cells in the CPM had few Sox9^+^ progenitor cells compared to the number of CNCs (CNC: 88.92%±2.24%; CPM: 25.77%±0.21%; *P* < 0.001) (Fig. 3d). On the other hand, Sox9 was also expressed in some muscle progenitor cells in the limb (Fig. 3e–h). At E10, we could not identify desmin^+^ muscle progenitor cells in the limbs, which showed Sox9 expression. At E12, we detected desmin^+^ muscle in the limbs, and some muscle cells showed Sox9 expression (Fig. 3f,g). The limb muscle anlage had few Sox9+ progenitor cells compared to the tendon and bone anlagen (muscle: 24.21%±7.18%; tendon and bone: 96.70%±0.75%; *P* < 0.01) (Fig. 3h). Therefore, Sox9 was expressed in some muscle progenitor cells *in vivo* (Fig. 3).

**Figure 4:**
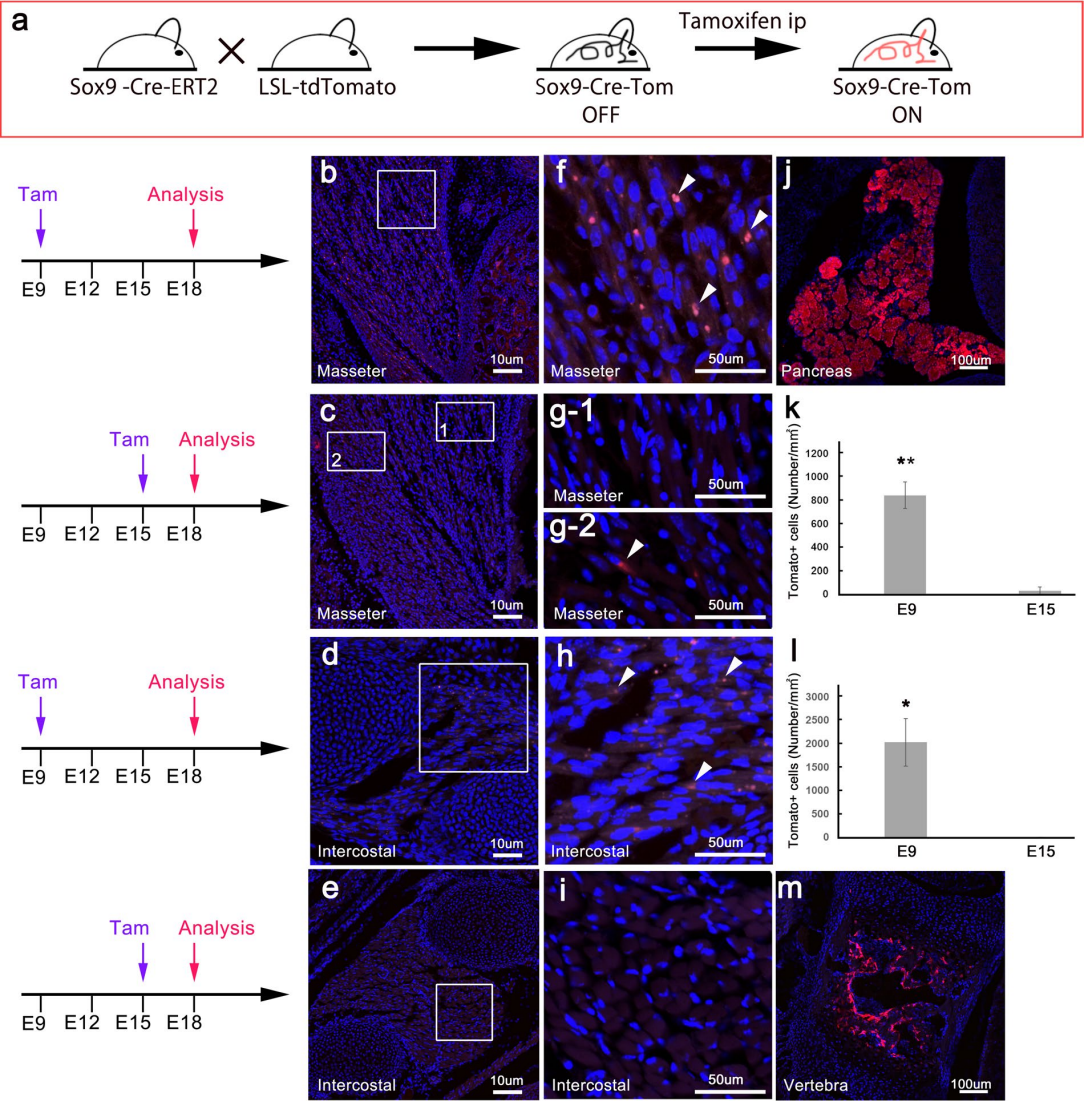
Lineage tracing of Sox9-expressing cells in developing muscle. (**a**) Schematic showing the generation of double-transgenic *Sox9*^*creERT2*^*/Rosa26-loxP-stop-loxP-tdTomato* reporter mice. We analyzed the (**b, c, f, g**) masseter muscle, (**d, e, h, i**) intercostal muscle, (**j**) pancreas, and (**m**) lumber vertebra. Mice were induced at (**b, d, f, h, j**) E9 and (**c, e, g, i, m**) E15 and analyzed at E18. The number of tdTomato^*+*^ cells in muscles induced at E9 and analyzed at E18 was much larger than that induced at E15 and analyzed at E18 (**k, i**; masseter: *P* < 0.01; intercostal: *P* < 0.05). Sox9, SRY-box containing gene 9.

To determine whether Sox9 is expressed in the muscle, we generated double-transgenic *Sox9*^*creERT2*^*/Rosa26-loxP-stop-loxP-tdTomato* reporter mice^19^. We detected the enrichment of tdTomato^+^ cells in the masseter and intercostal muscles of mice induced at E9 (Fig. 4b,f,d,h) and few tdTomato^+^ cells in the masseter muscle in mice induced at E15 (Fig. 4c,g). We could not identify tdTomato^+^ cells in the intercostal muscle in mice induced at E15 (Fig. 4e,i). The number of tdTomato^+^ cells in the masseter and intercostal muscles in mice induced at E9 was high compared to that in mice induced at E15 (masseter: *P* < 0.01, Fig. 4k; intercostal: *P* < 0.05, Fig. 4i). The tdTomato^+^ area was located between the nuclei of the muscle (Fig. 4f,h). In mice induced at E9, we could clearly identify tdTomato^+^ cells in the pancreas (Fig. 4j). The lumber vertebrae showed the enrichment of tdTomato^+^ cells in mice induced at E15 (Fig. 4m). These results clearly showed that Sox9 was expressed in muscle progenitor cells.

### Sox9 expression in muscle progenitor cells *in vitro*

**Figure 5:**
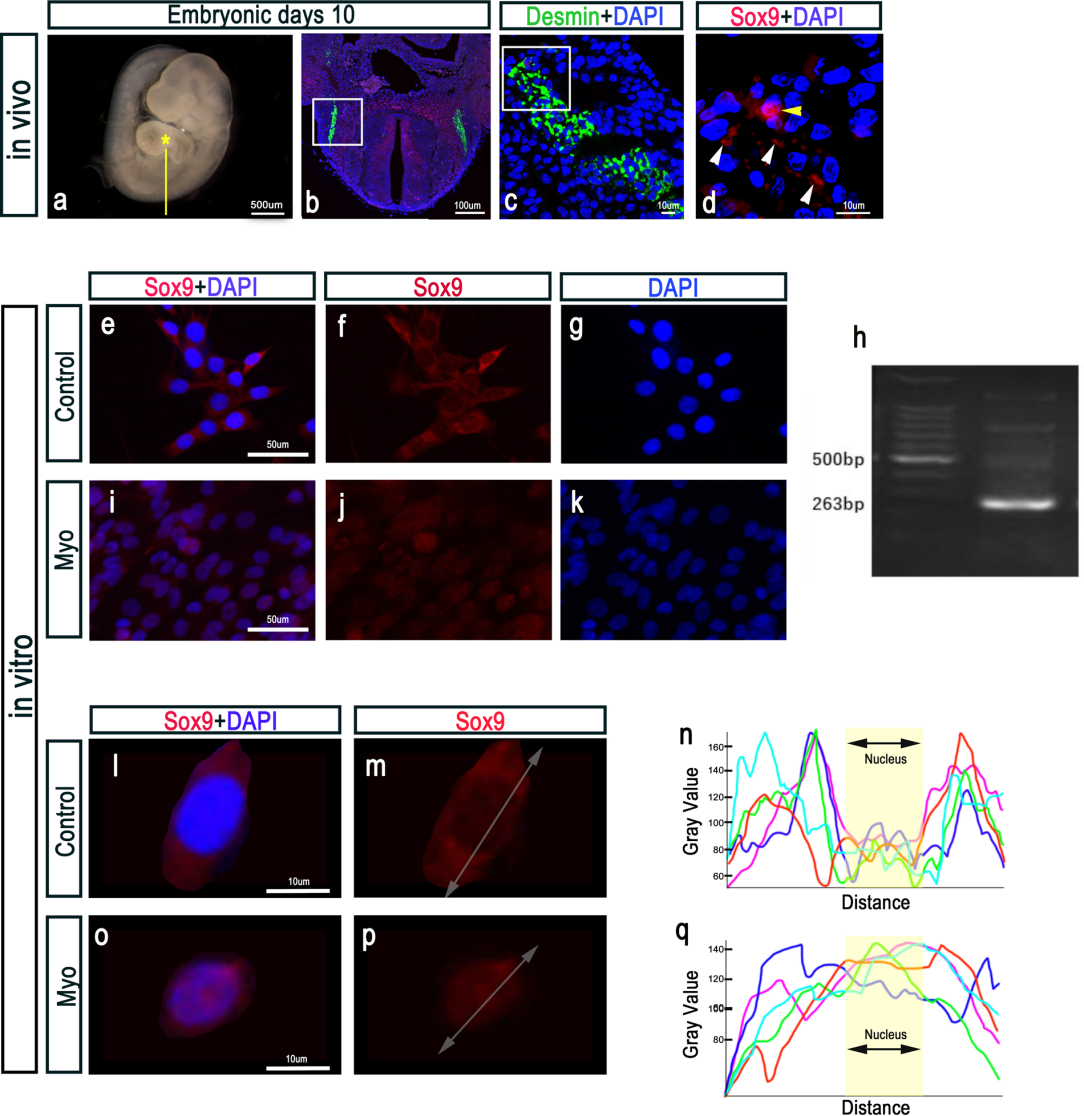
Sox9 expression *in vivo* and *in vitro*. (**a–d)** Desmin and Sox9 were expressed in the myotome at E10. (**e–g**) Sox9 expression in the control group (undifferentiated C2C12 cells). (**i–k**) Sox9^+^ progenitor cells under muscle induction (experimental group). (**l–q**) Single-cell analysis of C2C12 cells. (**l, m, o, p**) Immunohistochemical staining of Sox9 and (**n, q**) fluorescence intensity of Sox9 expression. (**a–d**) Sox9 was expressed in the cytoplasm of muscle progenitor cells. (**e-h**) In the control group, Sox9 was clearly expressed in the cytoplasm of C2C12 cells, and we identified Sox9 mRNA expression. (**l-n**) Immunofluorescence intensity of Sox9 in the cytoplasm was high compared to that in the nucleus. (**i-k**) In the experimental group, Sox9 was expressed in the nucleus of muscle progenitor cells. (**o-q**) Myogenic induction indicated Sox9 expression in the nucleus. Sox9, SRY-box containing gene 9; mRNA, messenger RNA.

We noticed that Sox9 was expressed in the cytoplasm of muscle progenitor cells in the myotome (Fig. 5a–d). Sox9 expression in sex cells translocates from the cytoplasm to the nucleus at the onset of male sexual differentiation^25^. To identify whether Sox9 expression during myogenesis translocates from the cytoplasm to the nucleus, we investigated the mouse myoblast C2C12 cell line. In the control group (undifferentiated C2C12 cells), Sox9 was clearly expressed in the cytoplasm (Fig. 5e–g,l,m). Reverse transcription-polymerase chain reaction (RT-PCR) showed Sox9 messenger RNA (mRNA) expression in the control group (Fig. 5h). In myogenic induction, Sox9 was expressed in the nucleus (Fig. 5i-k,o,p). In addition, in the control group, the immunofluorescence intensity of Sox9 in the cytoplasm was high compared to that in the nucleus (Fig. 5l–n). In contrast, myogenic induction resulted in Sox9 expression in the nucleus of C2C12 cells (Fig. 5o–q).

### Function of Sox9 in the development of the musculoskeletal system

**Figure 6:**
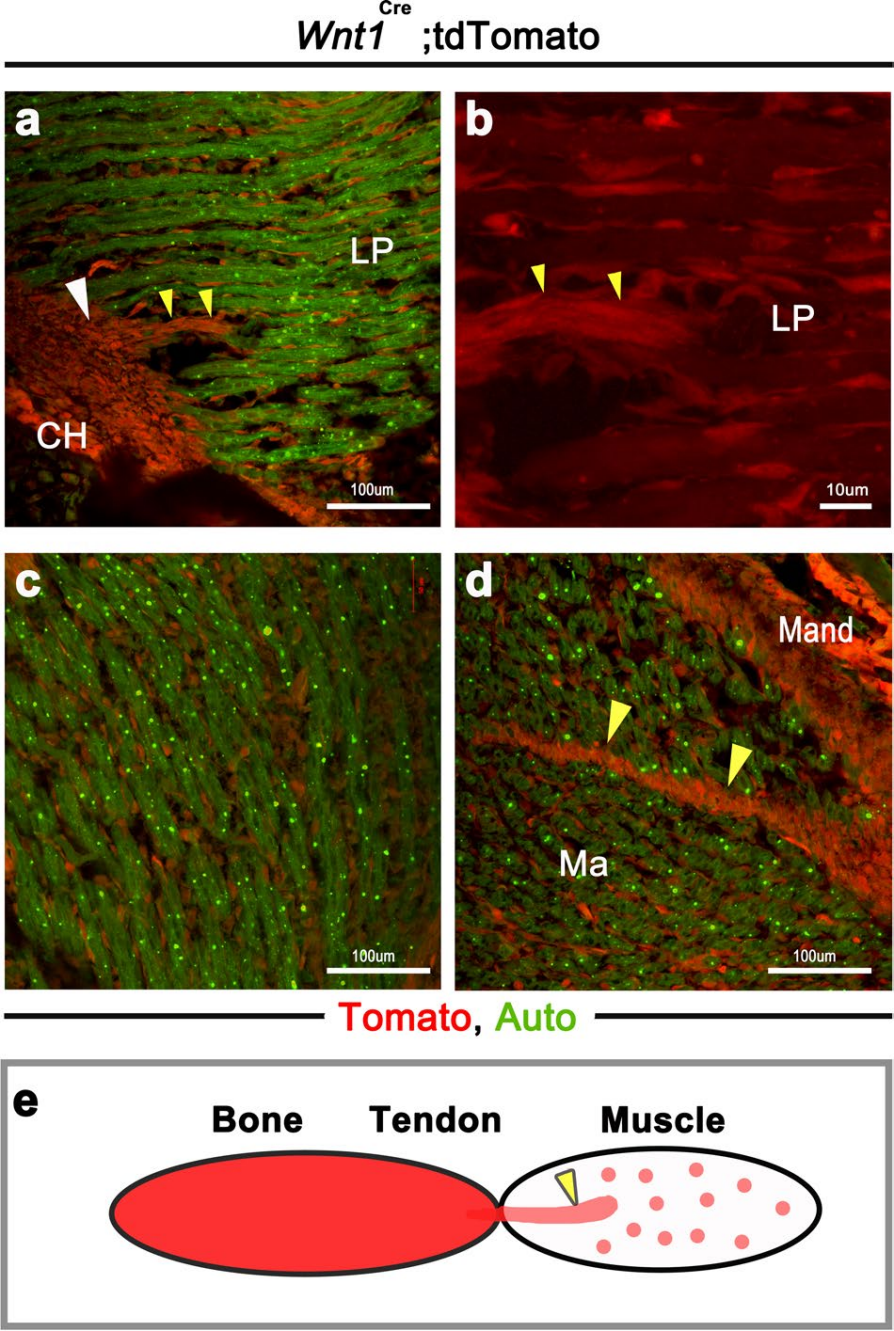
Wnt1 expression in each component of the musculoskeletal system. (**a**,**b**) The region of the lateral pterygoid muscle attachment to the mandibular condyle. (**c**) The fibers of the masseter muscle. (**d**) The region of the masseter muscle attachment to the mandible. (**b**) High-magnification view of panel (**a**). (**a-d**) Wnt1 was expressed in the muscle-associated connective tissue, tendon, and bone. (**e**) Wnt1-expressing region: bone, tendon, and muscle-associated connective tissue. White arrows, tendon; yellow arrows, intramuscular tendon; CH, condylar head; LP, lateral pterygoid muscle; Ma, masseter; Mand, mandibular bone.

The *Wnt1* gene plays important developmental roles in the CNC^4^. To identify *Wnt1* expression in the musculoskeletal system of the head, we used *Wnt1*^*Cre*^*;tdTomato* mice. Because muscle fibers exhibit a high level of green autofluorescence^26^, we could detect the muscle areas (Fig. 6a–d). tdTomato^+^ cells were observed throughout the muscle-associated connective tissue, tendon, and bone in the head (Fig. 6e) (Fig. 6a,b: the regions of lateral pterygoid muscle attachment to the condylar head; Fig. 6c and d: the regions of masseter muscle attachment to the mandible)^27,28^. Therefore, *Wnt1* was expressed throughout the musculoskeletal system in the head (Fig. 6e).

**Figure 7:**
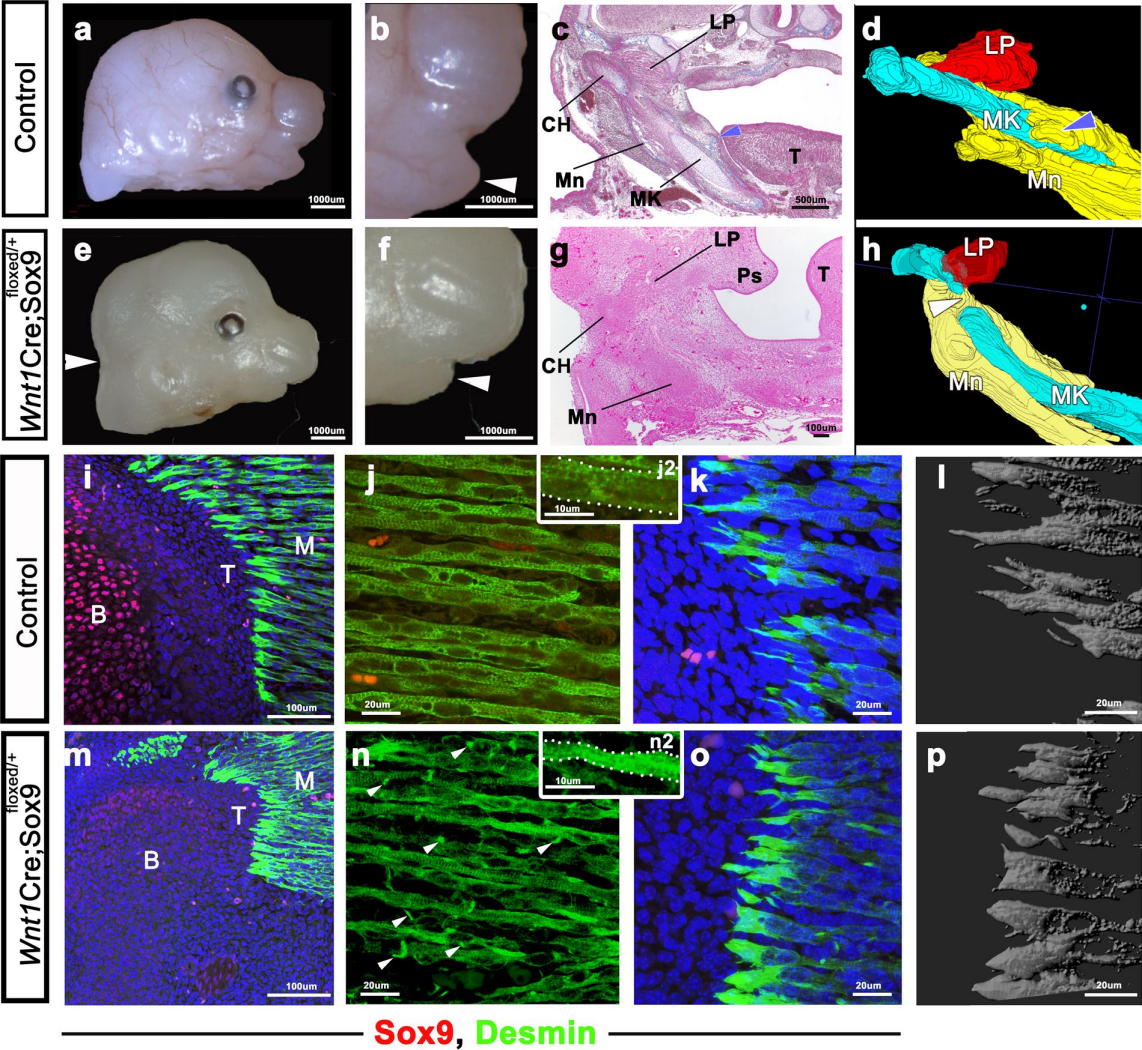
Function of Sox9 in the development of the musculoskeletal system. (**a–p**) Comparison of *WntCre;Sox9*^*floxed/+*^ mice with control mice. Lateral view of the head in (**a, b**) control and (**e, f**) *WntCre;Sox9*^*floxed/+*^ mice. (**c, g**) H&E staining and (**d, h**) 3D reconstruction of the mandibular bone and its surrounding structures. (**i–p**) Immunohistochemical staining of desmin (green) and Sox9 (red). Region of lateral pterygoid muscle attachment to the mandibular condyle in (**i**) control and (**m**) *WntCre;Sox9*^*floxed/+*^ mice. Muscle fibers in the lateral pterygoid muscle in (**j**) control and (**n**) *WntCre;Sox9*^*floxed/+*^ mice. MTJ in the lateral pterygoid muscle in (**k**) control and (**o**) *WntCre;Sox9*^*floxed/+*^ mice. (**l, p**) 3D reconstruction of the MTJ. *Wnt1-Cre;Sox9*^*flox/+*^ mice at E15.5 have a small lower jaw (**b, f**), a cleft secondary palate (**g**), and deformed Meckel’s cartilage (**d, h**, white arrowhead) and mandibular bone (**d, h**, blue arrowhead). (**i, m**) *Wnt1-Cre;Sox9*^*flox/+*^ mice have tendon and bone deformation in the TMJ. (**n**) Muscle fibers in *Wnt1-Cre;Sox9*^*flox/+*^ mice are narrow compared to those in control mice (**j**). The lateral pterygoid muscle in *Wnt1-Cre;Sox9*^*flox/+*^ mice has immature muscle cells (**n**, arrowheads). The muscle fiber ends of the MTJ in control mice are sharp, while the muscle fibers of the MTJ in *Wnt1-Cre;Sox9*^*flox/+*^ mice have comparatively rounded ends (**k, l, o, p**). Sox9, SRY-box containing gene 9; H&E, hematoxylin and eosin; B, bone; CH, condylar head; LP, lateral pterygoid muscle; M, muscle; MK, Meckel’s cartilage; Mn, mandibular bone; MTJ, myotendinous junction; T, tendon (**i,m**) or tongue (**c,g**); TMJ, temporomandibular joint.

To determine the functional role of Sox9 in the development of the musculoskeletal system, we examined the TMJ in *Wnt1*^*Cre*^*;Sox9*^*flox/+*^ mice^4^. *Wnt1*^*Cre*^*;Sox9*^*flox/+*^ mice at E15.5 had a small lower jaw, a cleft secondary palate, and deformed Meckel’s cartilage and mandibular bone (Fig. 7a–h). *Wnt1*^*Cre*^*;Sox9*^*flox/+*^ mice had muscle, tendon, and bone deformation in the TMJ (Fig. 7i–p). The formation of tendon and bone was significantly decreased in *Wnt1*^*Cre*^*;Sox9*^*flox/+*^ mice at E15.5 (Fig. 7I,m). In addition, the masticatory muscles of *Wnt1*^*Cre*^*;Sox9*^*flox/+*^ mice showed hypoplasia (Fig. 7j,n). Muscle fibers of the lateral pterygoid (one of the masticatory muscles) in *Wnt1*^*Cre*^*;Sox9*^*flox/+*^ mice were narrow compared with those in control mice (Fig. 7j,n). Immature muscle cells were located in the lateral pterygoid of *Wnt1*^*Cre*^*;Sox9*^*flox/+*^ mice (Fig. 7n, arrow). The muscle fiber ends of the MTJ in control mice were sharp, while they were comparatively rounded in *Wnt1*^*Cre*^*;Sox9*^*flox/+*^ mice (Fig. 7k,l,o,p).

## Discussion

Several studies have revealed the function of Sox9 in tendon and bone development^2–4,12–19^. Nevertheless, the fundamental question of whether Sox9 is expressed in developing muscles has been largely neglected. To reveal whether Sox9 controls the development of three components (muscles, tendons, and bones) of the musculoskeletal system, we studied the expression of Sox9 in developing muscle. This study demonstrated that muscle progenitors show Sox9 expression. Therefore, we revealed that Sox9 controls all the main components of the musculoskeletal system.

**Figure 8:**
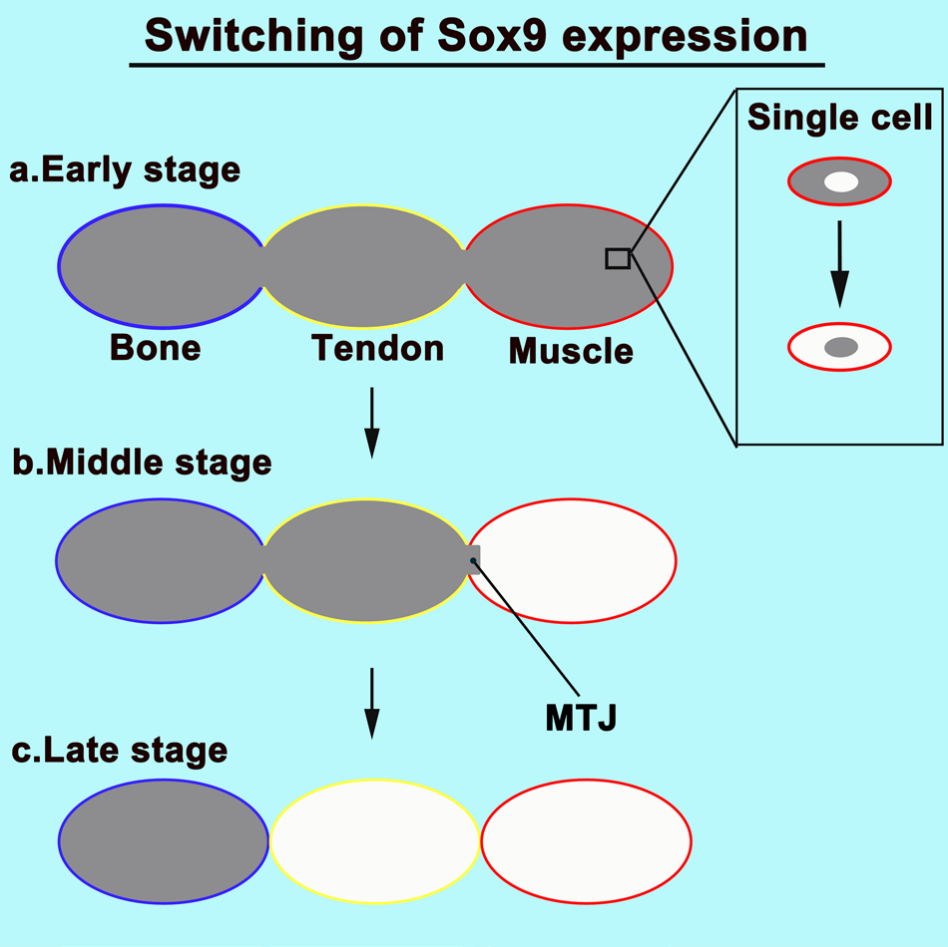
Schematic of switching of Sox9 expression. (**a**) Sox9 is expressed in progenitor cells of all components of the musculoskeletal system. (**b**) In the middle embryonic stage (E13), Sox9 was detected in the MTJ, tendon, and bone. (**c**) In the late embryonic stage (E16), bones show Sox9 expression. Therefore, it is essential to switch on Sox9 expression in each component (muscle, tendon, and bone) during the development of the musculoskeletal system. MTJ, myotendinous junction.

Our finding that the development of muscles, tendons, and bones is controlled by the switching of Sox9 expression provides new perspectives on the development of the musculoskeletal system. In the early stage, Sox9 was expressed in progenitor cells of all components of the musculoskeletal system. Subsequently, it was detected in the MTJ, tendon, and bone. In the late embryonic stage, bones showed Sox9 expression. Therefore, it is necessary to switch on Sox9 expression in each component (muscle, tendon, and bone) for the development of the musculoskeletal system (Fig. 8). We considered that the simplification of the functioning of transcription factors allows muscles, tendons, and bones to easily establish the complex structure of the musculoskeletal system.

It is well known that tendons and bones have the same origin. Tendons and bones are derived from the sclerotome, the lateral plate mesoderm, and the neural crest^2–5^. Tendon progenitor cells in the trunk develop from the syndetome, which is in the rear of the sclerotome^7,8^. In addition, in the sclerotome, Scx and Sox5 are coexpressed in the multipotent cell group, which can differentiate into cartilage and tendons, and are then expressed specifically in tendon and cartilage regions^7^. Sox9 and Scx were also detected in the subpopulations of tendon/ligament progenitor cells and chondroprogenitor cells^7,13^. In this study, muscle progenitor cells showed Sox9 expression both *in vivo* and *in vitro*. Therefore, the components of the musculoskeletal system (muscle, tendon, and bone) originate from Sox9^+^ progenitor cells. However, little is known about whether muscles, tendons, and bones have the same origin. This hypothesis is supported by the differentiation of somites into muscles, tendons, and bones^23^.

Previous studies on myoblastic cell lines in vitro have suggested that muscles, tendons, and bones have the same origin because they retain the capacity to differentiate into a chondrogenic, osteoblastic lineage or tenogenic lineage. According to Bettex-Galland and Wiesmann^29^, L6 myoblasts differentiate into chondrocytes under the influence of demineralized bone. Katagiri *et al*^20^. showed that bone morphogenetic protein-2 (BMP2) converts C2C12 cells into an osteoblast lineage. L6 myoblasts show Sox9 expression, which plays a role in chondrogenesis^21^. According to Uemura *et al*^30^., myostatin promotes tenogenic differentiation of C2C12 myoblastic cells. C2C12 and L6 appear to be stem cells that can differentiate into each component of the musculoskeletal system.

A few researchers have reported that muscle shows Sox9 expression. Rat L6 myoblastic cells show Sox9 expression, but after several days of culture, there is a decline in the level of Sox9^21^. We demonstrated that muscle progenitors show Sox9 expression *in vivo* and *in vitro* and that Sox9 expression in C2C12 cells translocate from the cytoplasm to the nucleus during muscle induction. Since Sox9 represses muscle gene expression^22^, myoblasts may retain their undifferentiated state. To retain an undifferentiated state, muscle progenitor cells expressed Sox9 in the cytoplasm (Fig. 8).

The *Wnt1* gene plays an important role in the development of the neural crest and its derivatives, and the *Wnt1*^*Cre*^ transgenic mouse line is widely used to investigate neural crest development. To clarify the role of Sox9 in the development of neural crest derivatives, Mori-Akiyama *et al*^4^. performed gross and histological observations using *Wnt1Cre;Sox9*^*flox/flox*^ mice. The analysis showed that all cartilage and endochondral bones, such as the anterior part of the cranial base, Meckel’s cartilage, the malleus, the incus, and the nasal capsule, were missing. Although the bone features of *Wnt1Cre;Sox9* ^*flox/flox*^ mice have been described^4^, little is known about the muscles and tendons of these mice. We found that *Wnt1Cre;Sox9*^*Flox/+*^ mice have a cleft palate, deficient Meckel’s cartilage, a small mandibular body, and hypoplastic condylar cartilage (Fig. 7). Moreover, these mice show hypoplasia of masticatory muscles and their tendons (Fig. 7). Because previous studies showed that Sox9 plays an important role in tendon and bone development^18,19^, it is easy to understand why tendon and bone hypoplasia is observed in *Wnt1Cre;Sox9*^*Flox/+*^ mice. The reason why muscle hypoplasia occurs in *Wnt1Cre;Sox9*^*Flox/+*^ mice is that defects in Sox9 expression in muscle connective tissues cause hypoplasia of muscle fibers (Figs. 6 and 7)^27,28^.

## Conclusion

Sox9 is expressed in progenitor cells of all components of the musculoskeletal system. However, the muscles and tendons do not express Sox9 during the late embryonic period. Therefore, it is essential to switch on Sox9 expression in each component (muscle, tendon, and bone) during the development of the musculoskeletal system (Fig. 8). In addition, a decrease in Sox9 expression in muscle-associated connective tissues, tendons, and bones leads to hypoplasia of the muscle, tendon, and bone. Therefore, Sox9 controls the development of each component of the musculoskeletal system.

## Methods

### Experimental animals

All experiments in mice were performed in accordance with the National Institutes of Health (NIH) guidelines for the care and use of animals. The experiments were also approved by the Tokyo Dental College Institutional Animal Care and Use Committee (protocol #240106). *Wnt1*^*Cre*^ (129S4. Cg-E2f1^Tg(Wnt1-cre)2Sor^/J), *Sox9*^flox/flox^ (B6.129S7-Sox9^tm2Crm^/J), *Sox9*^*creER*^ (STOCK Tg(Sox9^cre/ERT2^)1Msan/J), and *R26*^tdTomato^ (B6;129S6-Gt(ROSA)26Sor^tm14(CAG-tdTomato)Hze^/J) mice were purchased from Jackson Laboratory (Bar Harbor, ME, USA). All mice were bred under specific-pathogen-free conditions. *Wnt1*^*Cre*^ and *Sox9*^*creER*^ mice were mated with *R26*^tdTomato^ mice to generate *Wnt1*^*Cre*^; *R26*^tdTomato^ (*Sox9*^*creER*^; *R26*^tdTomato^) mice. *Wnt1*^*Cre*^ transgenic mice were mated with *Sox9*^*flox/flox*^ mice to generate *Wnt1*^*Cre*^; *Sox9*^flox/+^ mice. We performed PCR to genotype each strain according to the instructions of the Jackson Laboratory. A female mouse was housed with a male mouse overnight, and noon of the day when we observed the vaginal plug was designated as E0.5.

To trace the lineages of Sox9-expressing cells in developing muscle, we generated double-transgenic *Sox9*^*creERT2*^*/Rosa26-loxP-stop-loxP-tdTomato* reporter mice in whom Cre expression in Sox9^+^ progenitor cells could be induced at different developmental stages by administration of tamoxifen (Tam).

### Histological analysis

We fixed fetal tissues in 4% phosphate-buffered paraformaldehyde (PFA). When we made the paraffin blocks, we decalcified the specimens using 10% ethylenediaminetetraacetic acid (EDTA) for 7 days at room temperature. We prepared the paraffin blocks using standard methods and cut a series of 5-μm-thick tissue sections using a sliding microtome (Leica Biosystems, Wetzlar, Germany). When we made the frozen blocks, we incubated the tissue samples overnight in 30% sucrose in phosphate-buffered saline (PBS). Then, we embedded the tissue samples in Tissue-Tek OCT compound (Sakura Finetek, Japan). Next, we cut 15-μm-thick sections using a CM1950 cryomicrotome (Leica Biosystems, Wetzlar, Germany) and stained the sections with Hoechst 33342 (1:1000 dilution; Thermo Fisher Scientific, Waltham, MA, USA) to visualize the nuclei using an Axio Imager wide-field fluorescence microscope (Zeiss, Oberkochen, Germany). We analyzed the images using ImageJ (NIH, Bethesda, MD, USA). For 3D reconstruction, we loaded the digital images of the hematoxylin and eosin (H&E)-stained serial sections into Amira (Visage Imaging, Inc., Richmond, Australia) by using a voxel size appropriate for the section thickness.

### Immunohistochemical analysis

We incubated the sections overnight at 4 °C with the following primary antibodies: mouse anti-desmin antibody (1:1000 dilution; Merck Millipore, Burlington, MA, USA) and rabbit anti-sox9 antibody (1:1000 dilution; Merck Millipore). Then, we stained them for 1.5 h at room temperature using the ABC staining kit (Funakoshi, Tokyo, Japan) and the following secondary antibodies: donkey anti-mouse immunoglobulin G (IgG) Alexa Fluor 488 (1:1000 dilution; Thermo Fisher Scientific) and donkey anti-goat IgG Alexa Fluor 555 (1:1000 dilution; Thermo Fisher Scientific). We treated a few sections with ImmPACT 3,3-diaminobenzidine (DAB) (Funakoshi, Tokyo, Japan) to detect any reactions and then inspected the sections after counterstaining with hematoxylin.

### Double-staining of ALP and desmin

We stained the sections using an ALP staining kit (Primary Cell, Hokkaido, Japan) according to the manufacturer’s instructions. Briefly, we rinsed the sections in running distilled water for 1 min, after which we added 50 mL of staining solution dropwise to each section. Then, we incubated the sections for 3 h at room temperature until the ALP staining was a bright intense blue, and then we washed the sections with PBS. Next, we incubated them in 3% hydrogen peroxide with methanol for 30 min, subjected them to several additional washings with PBS, and then incubated them in 3% bovine serum albumin for 1 h to block nonspecific binding. Subsequently, we treated the sections with a primary antibody against desmin (1:1000 dilution; Abcam, Cambridge, UK) and incubated them overnight in a moisture chamber at 37 °C. Then, we applied a secondary antibody using EnVision^TM^ + Dual Link System-horseradish peroxidase (HRP; Dako, Tokyo, Japan) at room temperature. Finally, after several more washes with PBS, we treated the sections with ImmPACT DAB (Funakoshi) to detect any reaction and then inspected them after counterstaining with hematoxylin.

### RNA in situ hybridization

The anti-sense probe for *Scx* has been described previously^31^. We labeled the probe with digoxigenin (DIG RNA labeling mix; Roche, Rotkreuz, Switzerland) and performed hybridization by following a standard protocol. Briefly, we fixed the sections for 10 min using 4% PFA, digested them using 1 μg/mL proteinase K (Roche) for 5 min, and then fixed them again for 5 min. Next, we performed acetylation for 10 min in a solution containing triethanolamine, hydrochloric acid, and acetic anhydride. We preblocked the sections using hybridization buffer (50% formamide, 5x saline sodium citrate [SSC], 50 μg/mL yeast tRNA, 1% sodium dodecyl sulfate [SDS], and 50 μg/ml heparin) and subsequently incubated them with an *Scx* anti-sense probe diluted to 1 ng/μL in hybridization buffer. After washing away the unbound probes using SSC buffer, we detected the probes in the sections using antidigoxigenin antibody conjugated to ALP (Roche) and BM purple (Roche).

### Tamoxifen treatment

We dissolved tamoxifen (T5648; Sigma-Aldrich, St. Louis, MO, USA) in ethanol and then diluted it in corn oil (C8267; Sigma-Aldrich) at a concentration of 10 mg/mL, as described previously^32^. Then, we injected 1.5 mg or 3 mg of tamoxifen into the peritoneal cavity of pregnant mice at E9 or E15, respectively, and coinjected 1 mg/40 g of progesterone (P8783; Sigma-Aldrich).

### C2C12 cell differentiation

We maintained C2C12 cells in Dulbecco’s Modified Eagle Medium (DMEM; Mediatech) containing 10% (vol/vol) fetal bovine serum (Tissue Culture Biologicals, Tulare, CA, USA) and incubated them in a 5% CO_2_ atmosphere at 37 °C. Then, we differentiated 95%–100% confluent C2C12 myoblasts into myotubes using 2% (vol/vol) horse serum (J. R. Scientific, Woodland, CA, USA) in DMEM. We maintained the C2C12 cells in 2% horse serum–containing DMEM and changed the medium every second day until the C2C12 cells fully differentiated into myotubes, which required approximately 4–6 days (muscle induction group). In the control group, 95-100% confluent C2C12 myoblasts were maintained in DMEM containing 10% (vol/vol) FBS and incubated at 37 °C with 5% CO2 for 4-6 days.

Cells were washed with warm PBS (37 °C), fixed in 4% paraformaldehyde at room temperature for 30 min and then subjected to immunofluorescence staining for myotubes and 4,6-diamidino-2-phenylindole (DAPI) staining for nuclei. The primary antibody was rabbit anti-sox9 antibody (1:1000, Merck Millipore), and the secondary antibody was donkey anti-goat IgG Alexa Fluor 555 (1:1000, Thermo Fisher Scientific). To verify the validity of the immunohistostaining results, we performed RT-PCR on the control group (undifferentiated C2C12 cells).

### Statistical analysis

All statistical analyses were performed by using SPSS Statistics 21.0 (IBM, Armonk, NY, USA). *P*-values were calculated using Student’s *t*-test. The between-group differences for which *P* < 0.05 were considered statistically significant (*P < 0.05; **P < 0.01; ***P < 0.001 are used throughout the report). Error bars show the standard deviation of the mean.

## Data Availability

All data generated or analyzed during this study are included in this published article.
